# MRI-based radiomic prognostic signature for locally advanced oral cavity squamous cell carcinoma: development, testing and comparison with genomic prognostic signatures

**DOI:** 10.1186/s40364-023-00494-5

**Published:** 2023-07-16

**Authors:** Anna Corti, Loris De Cecco, Stefano Cavalieri, Deborah Lenoci, Federico Pistore, Giuseppina Calareso, Davide Mattavelli, Pim de Graaf, C. René Leemans, Ruud H. Brakenhoff, Marco Ravanelli, Tito Poli, Lisa Licitra, Valentina Corino, Luca Mainardi

**Affiliations:** 1 Department of Electronics, Information and Bioengineering, Politecnico di Milano, Milan, Italy; 2 Integrated Biology of Rare Tumors, Department of Research, Fondazione IRCCS, Istituto Nazionale dei Tumori, Milan, Italy; 3 Head and Neck Medical Oncology Department, Fondazione IRCCS, Istituto Nazionale dei Tumori, Milan, Italy; 4 Department of Oncology and Hemato-Oncology, Università degli studi di Milano, Milan, Italy; 5 Radiology Department, Fondazione IRCCS, Istituto Nazionale dei Tumori, Milan, Italy; 6 Unit of Otorhinolaryngology-Head and Neck Surgery, Department of Medical and Surgical Specialties, Radiological Sciences, and Public Health, ASST Spedali Civili of Brescia, University of Brescia, Brescia, Italy; 7 Amsterdam UMC location Vrije Universiteit, Radiology and Nuclear Medicine, de Boelelaan 1117, Amsterdam, The Netherlands; 8 Cancer Center Amsterdam, Imaging and Biomarkers, Amsterdam, The Netherlands; 9 Amsterdam UMC location Vrije Universiteit, Otolaryngology-Head and Neck Surgery, de Boelelaan 1117, Amsterdam, The Netherlands; 10 Unit of Radiology, Department of Medical and Surgical Specialties, Radiological Sciences, and Public Health, ASST Spedali Civili of Brescia, University of Brescia, Brescia, Italy; 11 Maxillo-Facial Surgery Division, Head and Neck Department, University Hospital of Parma, Parma, Italy; 12 Cardiotech Lab, Centro Cardiologico Monzino IRCCS, Milan, Italy

**Keywords:** Radiomics, Radiogenomics, Magnetic resonance imaging, Head and neck cancer, Survival models, Overall survival, Oral cavity squamous cell carcinoma

## Abstract

**Background:**

. At present, the prognostic prediction in advanced oral cavity squamous cell carcinoma (OCSCC) is based on the tumor-node-metastasis (TNM) staging system, and the most used imaging modality in these patients is magnetic resonance image (MRI). With the aim to improve the prediction, we developed an MRI-based radiomic signature as a prognostic marker for overall survival (OS) in OCSCC patients and compared it with published gene expression signatures for prognosis of OS in head and neck cancer patients, replicated herein on our OCSCC dataset.

**Methods:**

For each patient, 1072 radiomic features were extracted from T1 and T2-weighted MRI (T1w and T2w). Features selection was performed, and an optimal set of five of them was used to fit a Cox proportional hazard regression model for OS. The radiomic signature was developed on a multi-centric locally advanced OCSCC retrospective dataset (n = 123) and validated on a prospective cohort (n = 108).

**Results:**

The performance of the signature was evaluated in terms of C-index (0.68 (IQR 0.66–0.70)), hazard ratio (HR 2.64 (95% CI 1.62–4.31)), and high/low risk group stratification (log-rank *p* < 0.001, Kaplan-Meier curves). When tested on a multi-centric prospective cohort (n = 108), the signature had a C-index of 0.62 (IQR 0.58–0.64) and outperformed the clinical and pathologic TNM stage and six out of seven gene expression prognostic signatures. In addition, the significant difference of the radiomic signature between stages III and IVa/b in patients receiving surgery suggests a potential association of MRI features with the pathologic stage.

**Conclusions:**

Overall, the present study suggests that MRI signatures, containing non-invasive and cost-effective remarkable information, could be exploited as prognostic tools.

**Supplementary Information:**

The online version contains supplementary material available at 10.1186/s40364-023-00494-5.

## Background

Oral cavity squamous cell carcinomas (OCSCCs), including tongue cancers, are the most common malignancies of the oral cavity, accounting for approximately half of all head and neck squamous cell carcinomas (HNSCCs) [1].

The treatment mainstay for loco-regionally advanced (i.e., clinical tumor-node-metastasis (cTNM) stage III/IVa-b according to the 8th edition of the AJCC/UICC staging system) OCSCC is surgery followed by adjuvant radiation, plus concomitant chemotherapy in case of adverse pathologic factors (i.e., residual tumor after resection and/or extracapsular spread to regional lymph nodes). In this scenario, the strongest prognostic factor currently available is pathologic tumor-node (pTNM) metastasis stage.

However, the pTNM is available only after performing surgery, and at diagnosis no robust baseline prognostic factors are available, with the only exception of the cTNM, that is obtained through clinical and radiological assessments. In this setting, magnetic resonance imaging (MRI) provides a high-quality resolution in determining soft tissue infiltration, so it is one of the most relevant tools to guide surgical planning. Nevertheless, so far images per se have not been used in the clinical practice for prognostic purposes, apart from determining the cTNM.

Thus, additional biomarkers are needed to better stratify patients, refine the stage-based clinical decision and lead to more personalized therapies. In this context, high throughput “omics” technologies have recently gained increasing interest for the identification of prognostic factors [2]. Among those, genomics and radiomics are the ones that have been more extensively investigated as far as the development of prognostic models is concerned [3–8].

Starting from early 2000, omics techniques including different comprehensive analyses on gene expression (transcriptomics), proteins and metabolites, were applied to identify biomarkers (see glossary in a very recent radiomic review [9]). In a review considering publications up to 2019 about transcriptomics and epigenomics in HNSCC, we reported 33 studies analyzing gene expression and disclosing biological and/or prognostic roles in the disease [10]. Thereafter, thanks to the availability of data from The Cancer Genome Atlas [11], gene expression signatures have been generated including all HNSCC anatomical subsites or specifically OCSCC [10, 12, 13].

Radiomics, namely the extraction and mining of quantitative features from radiological imaging, has the potential to provide non-invasive prognostic biomarkers. To date, several studies applied radiomics to characterize tumors, predict pathological features and predict clinical outcomes in HNSCC patients, as extensively reviewed in [14–16]. Most of the radiomic studies focusing on prognosis of overall survival (OS) in HNSCC patients were based on computer tomography or positron emission tomography [6, 7, 17–31], while only few prognostic models with features extracted from MRI were developed [32–42]. In particular, to the best of the authors’ knowledge, only three studies [32, 34, 42] developed an MRI-radiomic signature for prognosis of OS specific for OCSCC patients. Few other MRI-based radiomic studies focused on oral cavity cancer were proposed to predict tumor grading [43], pathological differentiation [44] and extracapsular nodal spread [45]. In this scenario, given the high heterogeneity of HNSCC patients [46] and the need for more personalized treatments, the development of MRI-based radiomic signature specific for OCSCC patients is fundamental.

To this aim we exploited our recently obtained database BD2Decide [47] that could enable linking together rigorously annotated patient-specific multiparameter clinical, pathologic, demographic, transcriptomics and radiomics data from the currently largest cohort of patients with locoregionally advanced HNSCC.

The purpose of this study was to develop a prognostic MRI-based radiomic signature from the retrospective OCSCC cohort, and to test it on the prospective one. Finally, the prognostic effect of the MRI-based radiomic model was compared to conventional prognostic metrics (cTNM and pTNM) and to some published gene expression prognostic signatures [48–54].

## Materials and methods

### Analyzed dataset

**Fig. 1:**
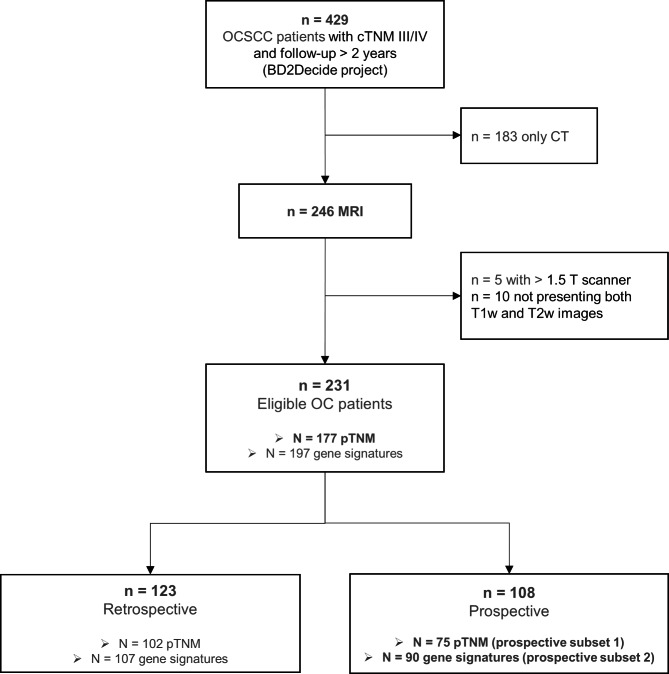
CONSORT flow diagram

A subset of the BD2Decide project (NCT02832102) patients with positive pathologic diagnosis of OCSCC and loco-regionally advanced disease (cTNM III, IVa or IVb according to the 8th edition of AJCC/UICC) treated with curative intent [47] was used for this study. The protocols were approved by the Ethical Committees of the participating centers, and data acquisition followed the General Data Protection Regulation of the EU. All patients signed the informed consent. The inclusion criteria of the study were the following: (i) availability of T1-weighted (T1w) and T2-weighted (T2w) MR image sequence and (ii) images acquired with 1.5 T scanner (Fig. 1). Images from the selected patients were collected from four different clinical centers: the Fondazione IRCCS Istituto Nazionale dei Tumori of Milan, Italy; the Azienda Ospedaliero Universitaria di Parma, Italy; the Spedali Civili di Brescia, Italy; the Amsterdam VU Medisch Centrum Medical Center, the Netherlands.

### Clinical endpoint

The clinical endpoint analyzed in this study was OS, defined as the time between the primary tumor diagnosis and the day of death or last follow-up. In details, OS times were calculated in months from the date of diagnosis to the date of death of any cause (event), and censored at the date of last follow-up for patients that were still alive.

### MRI image acquisition

For each patient T1w and T2w MRI images were acquired using scanners with a field strength of 1.5 T. The T1w and T2w images were acquired using a turbo spin-echo pulse sequence. Other image acquisition parameters, such as time of repetition, time of echo, pixel spacing, slice thickness, were not standardized. Table 1 gives an overview of the image acquisition parameters used to acquire the images in the study.

**Table 1:** Image acquisition parameters for the patients of the BD2Decide dataset

Image type	T1w	T2w
Time of repetition (ms)	486 [474–580]	4420 [3760–5300]
Time of echo (ms)	12 [9-12]	109 [107–110]
Pixel spacing (mm)	0.63 [0.59–0.69]	0.57 [0.56–0.61]
Slice thickness (mm)	3 [3-3.5]	3 [3-4]
Spacing between slices (mm)	3.9 [3.9–4.01]	3.9 [3.9–4.4]

Data are displayed by image sequence: T1-weighted (T1w); T2-weighted (T2w).

### Image segmentation

**Fig. 2:**
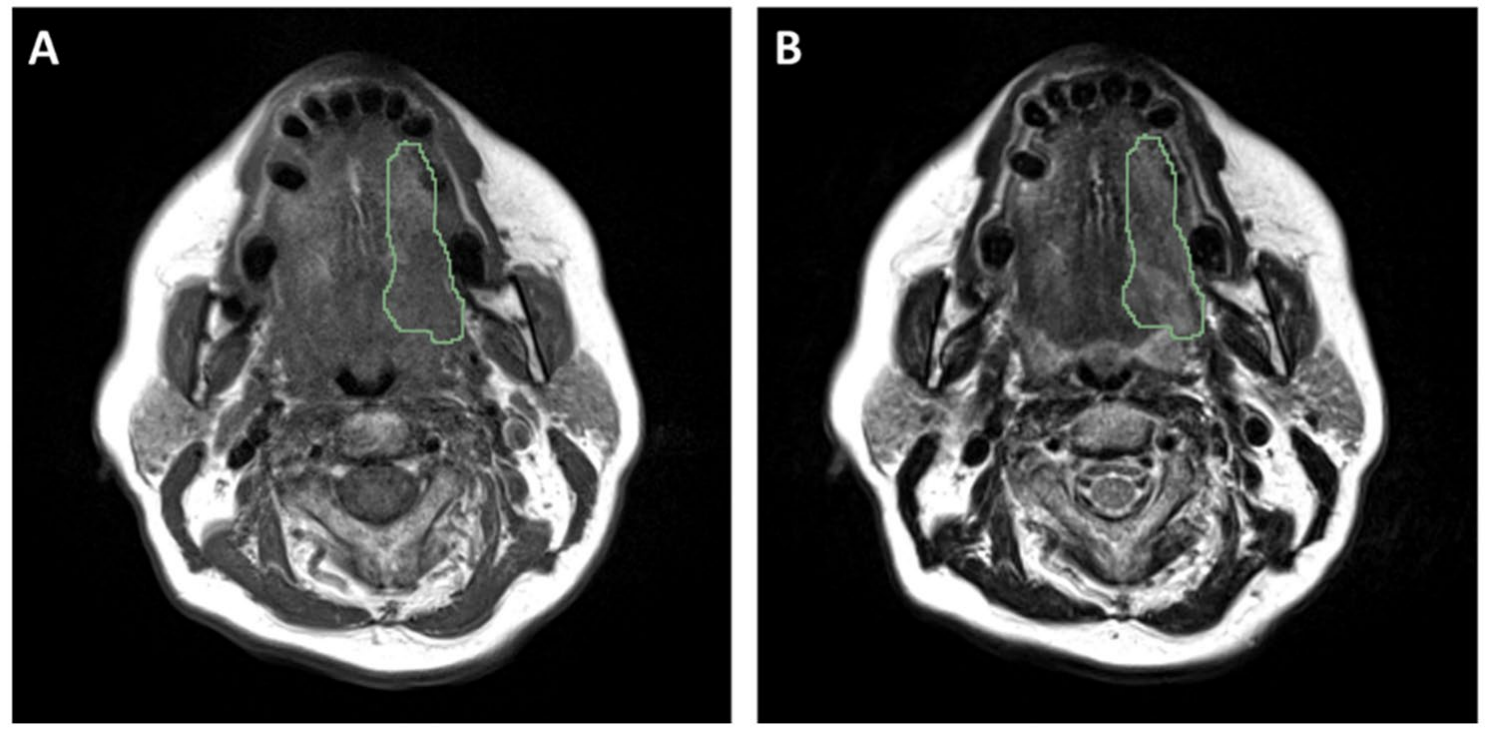
Type of magnetic resonance images acquired for the study: (**A**) T1-weighted image; (**B**) T2-weighted image. The segmented region of interest is also displayed

The gross tumor volume was segmented at the clinical centers using a semi-automatic segmentation software based on coupled shape modeling [55]. The segmentation of the region of interest (ROI), corresponding to the primary tumor, was performed manually slice by slice by expert radiologists (one for each center) dedicated to head and neck cancers. The tumor boundary was delineated by considering T2w sequence as reference, and checked and corrected based on the T1w sequence. An example of segmented images is displayed in Fig. 2.

### Image preprocessing

Image preprocessing was applied to the MRI images to reduce all the imaging-related sources of variability. Specifically, four steps of image preprocessing were applied [56]: (i) a 3D Gaussian filter with a 3 × 3 × 3 voxel kernel and σ = 0.5 was used to denoise the images; (ii) the N4ITK algorithm [57] was used for the correction of intensity non-uniformities due to local variations of the magnetic field; (iii) intensity standardization was performed using Z-score to ensure that each MRI image had similar ranges of signals; (iv) voxel size resampling to an isotropic resolution of 2 mm was performed with B-spline interpolation [58].

### Radiomic features extraction

The extraction of radiomic features was performed using Pyradiomics 2.2.0 (open-source, available at https://github.com/Radiomics/pyradiomics and run on Python) [59]. A total of 1072 radiomic features, 536 per image type (T1w, T2w) were extracted. The features belonged to different categories: shape and size (14 features), first order statistics (18 features), textural (40 features), wavelet (464 features). Textural features were computed using the grey level co-occurrence matrix (GLCM) and the grey level run length matrix (GLRLM). The full list of radiomic features is available in Pyradiomics documentation [60]. A fixed-bin histogram discretization (32 bins) was used prior to features extraction.

### Radiomic features postprocessing and radiomic model development

Features selection and survival model training was performed on the 123 retrospective patients (training dataset). First, features were Z-score normalized to ensure comparable ranges for the feature values. The mean and standard deviation used for the feature-wise normalization of the training set were then applied to normalize the features of the prospective set. The normalization was performed to improve the convergence of the optimization algorithms used during model fitting [61]. Then, as the number of extracted features was much larger than the number of available patients, a process of features selection was required to reduce the dimensionality of the radiomic dataset and avoid overfitting.

**Fig. 3:**
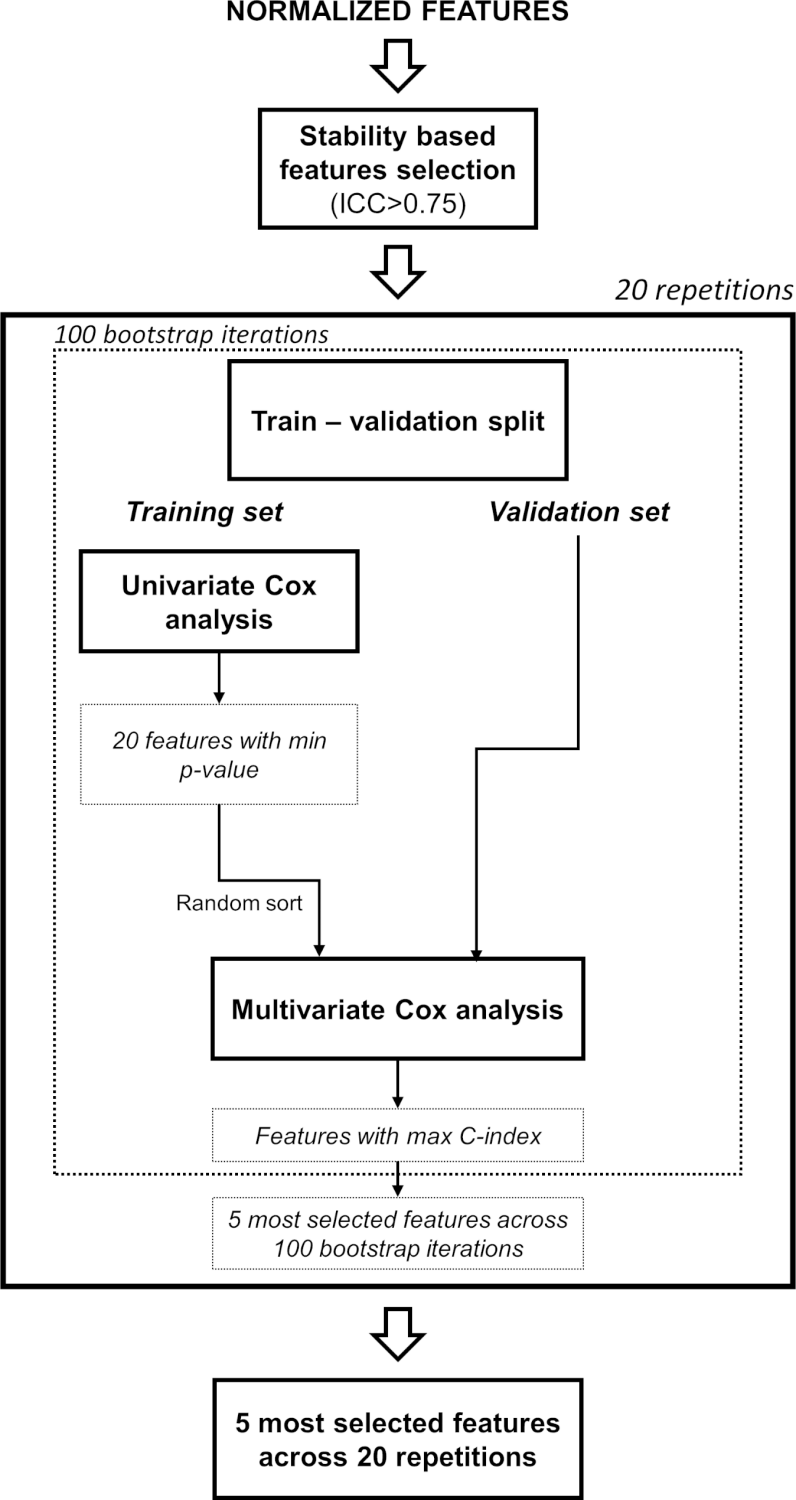
Features selection process

The features selection process was performed on the training set and comprised (i) stability analysis and (ii) supervised feature selection based on both univariate and multivariate Cox regression, similarly to a previous MRI-based radiomic study [62]. As regards the first step of the features selection, the stability of the features to small translations of the ROI (i.e., ± 10% of the length of the bounding box surrounding the ROI along both the x and y directions), used to mimic the effect of inter-reader variability in the segmentation, was evaluated as described elsewhere [56, 62, 63]. Features with intra-class correlation coefficient (ICC) above 0.75 were considered stable and selected. The second step of features selection consisted in the evaluation of features prognostic significance and Harrel’s concordance index (C-index) [64] in both univariate and multivariate Cox regression. Specifically, 100 bootstrap iterations were performed in which the training dataset was divided in independent subsets of training and validation (70% and 30%, respectively) and, at each iteration, potentially different feature sets can be found. For each iteration, univariate Cox models were used to select the features that were significantly prognostic for OS (*p* < 0.05). The 20 features associated with lower *p* in the univariate analysis were combined in a multivariate Cox model using a wrapper forward feature selection algorithm [62, 65]. The combination of features associated with the highest C-index was the selected feature set of the specific bootstrap iteration. At the end of the 100 bootstrap iterations the 5 most selected features were considered. The overall process (i.e., second step of features selection) was repeated 20 times with different seeds of the random number generator to increase the robustness, and the final features set contained the 5 most selected features over the 20 repetitions. Figure 3 schematically describes the 2-step process of features selection.

The final feature set was used to train a multivariate Cox model for OS on the retrospective set. The radiomic signature was thus obtained for each patient as the linear combination of the features and the corresponding regression coefficients. The median value of the signature in the training set was used as a threshold to classify the patients into high and low risk groups. Patients with a signature higher than a threshold are classified as high-risk patients, while patients with signature below the threshold are classified as low-risk patients.

All the steps of features postprocessing, feature selection and model training were performed in Matlab 2022a (Mathworks, Natick, MA, USA).

### Model testing and comparison

The independent prospective set comprised 108 patients. First the Z-score normalization of the features of the prospective set was performed, based on the mean and standard deviation of Z-score normalization estimated from the retrospective set. The radiomic signature of the prospective set was computed as linear combination of the features and the corresponding regression coefficients of the trained Cox model, and high/low risk classification was based on the threshold estimated from the training set (i.e., median value of the signature on the training set).

The radiomic signature prognostic performance was evaluated on both the retrospective (used for model training) and prospective (used for model testing) sets. First, C-index between the signature and the OS [64] was computed through 100 bootstrap iterations to obtain a distribution of C-indexes. Second, the hazard ratio (HR) was computed. Third, the *p*-value of the log-rank test [66] comparing the Kaplan-Meier curves [67] for high and low risk groups was evaluated.

The prognostic performance of the radiomic signature was compared with that of the cTNM and the pTNM on the prospective subset 1 (n = 75 patients as shown in Fig. 1), and with that of 7 published prognostic gene expression signatures (detailed below) on the prospective subset 2 (n = 90 patients as shown in Fig. 1). Finally, the relationship of the radiomic signature with the pTNM was also evaluated, by analyzing the distribution of the radiomic signature at different pTNM stages in the dataset of 177 patients (i.e., retrospective and prospective patients for whom the pTNM was available, Fig. 1).

### HNSCC/OCSCC gene expression signatures

Formalin-fixed, paraffin-embedded samples of prospective subset 2 (n = 90) were collected, and after histopathological revision, selected tumor areas were manually macro-dissected. RNA extraction was performed using the Qiagen RNeasy Mini Kit with the QIAcube robotic station (Qiagen, Düsseldorf, Germany), according to the manufacturer’s recommendations. Quantification and quality check were performed using the Qubit 3.0 fluorometer (Life Technologies, Carlsbad, CA, USA) and the TapeStation 4200 system (Agilent Technologies, Santa Clara, CA, USA).

Affymetrix human Clariom D arrays (Affymetrix, Santa Clara, CA, US) were used for the gene expression profiling in BD2decide cohort, targeting 540,000 transcripts. Probe synthesis was performed from total RNA using the GeneChip WT Pico Reagent Kit and WT Labeling Kit (Affymetrix) [68]. A total of 6 cycles pre-in vitro transcription amplification was performed according to the manufacturer’s protocol. Biotinylated and fragmented single-stranded cDNAs were hybridized to the arrays that were washed and stained using an FS-450 fluidics station (Affymetrix, fluidics protocol FS450_0001). Signal intensities were detected by a 30007G gene array scanner. The scanned images were processed using the Affymetrix GeneChip Command Console software. CEL data files were processed with Transcriptome Analysis Console Software v4.0.1 and further filtering procedures were performed in R software (version 4.0.3).

A survey of literature was performed to retrieve gene expression signatures generated for the prognosis in HNSCC/OCSCC tumor cohorts and subsequently reproduce the signatures’ score on the OCSCC prospective subset 2 (n = 90 patients). First, we created a database of signatures and the following inclusion criteria were applied: (i) paper analyzing whole gene expression data; (ii) paper reporting prognostic gene expression signatures; (iii) complete description of the bioinformatics methods; (iv) availability of the gene list, weights, and algorithm to compute signature’s score. Inclusion criteria were applied for a survey in literature in public database such as “Pubmed” (www.ncbi.nlm.nih.gov/pubmed) and “EMBASE” (www.embase.com) imposing a selection using keywords. Filters includes MeSH terms such as “HNSCC”, “signatures”, and “gene expression”. Consequently, each paper was carefully evaluated to exclude those based on proteins or immunohistochemistry. The genes were re-annotated based on EntrezID [69] and the bioinformatics methods were retrieved from the original manuscripts to reproduce the signature’s algorithms allowing applying them on external datasets along with thresholds for patient stratification.

### Statistical analysis

The characteristics of retrospective and prospective cohorts were compared using Mann-Whitney U tests for continuous variables and chi-squared test for categorical variables. Results of the Cox proportional hazard regression model are presented as Harrel’s C-index (median and IQR), HR (median and 95% confidence intervals (CIs)) and p-value of the log-rank test. To compare the distributions of C-index the Mann-Whitney U test was applied in case of comparison between two groups and the Kruskal-Wallis test with Tukey-Kramer p-value correction was applied in case of multiple comparison. All the statistical analyses were performed in Matlab 2022a.

## Results

### Patient characteristics

A dataset of 231 patients affected by stage III/IVa-b OCSCC, included in the BD2Decide database [47] for whom an MRI evaluation was available was considered for this study. The patient dataset comprised 123 retrospective patients and 108 prospective ones. Table 2 shows the clinical data of the selected patients included in the study.

The two sets, beside the expected difference in median follow-up, significantly differed in the higher percent of stage IVa-b and smoking status in prospective patients.

**Table 2:** Clinical data of the patients used for the study

Patient characteristics	Retrospective set (123)	Prospective set (108)	p-value
Date of diagnosis	2008–2014	2015–2018	-
Median follow-up	37.27 months (IQR 14.54–65.69)	25 months (IQR 16.91–31.56)	< 0.001
Gender			
*M* *F*	70 (57%) 53 (43%)	65 (60%) 43 (40%)	0.6884
Median age	61 years (IQR 54.25–69)	60 years (IQR 50–72)	0.8574
cTNM 8th edition			
*III* *IVa/b*	36 (29%) 87 (71%)	18 (17%) 90 (83%)	0.0291
Smoking status			
*Current/Former* *Never* *Unknown*	83 (67%) 40 (33%) 0	46 (43%) 37 (34%) 25 (23%)	< 0.001
Treatment			
*Including surgery* *Not including surgery*	107 (87%) 16 (13%)	98 (91%) 10 (9%)	0.41

Quantitative variables are displayed as median and interquartile range (IQR). Statistical tests (Mann-Whitney, chi-squared) are used to evaluate potential statistical differences between the patient cohorts.

### Features selection and survival model training and testing

**Fig. 4:**
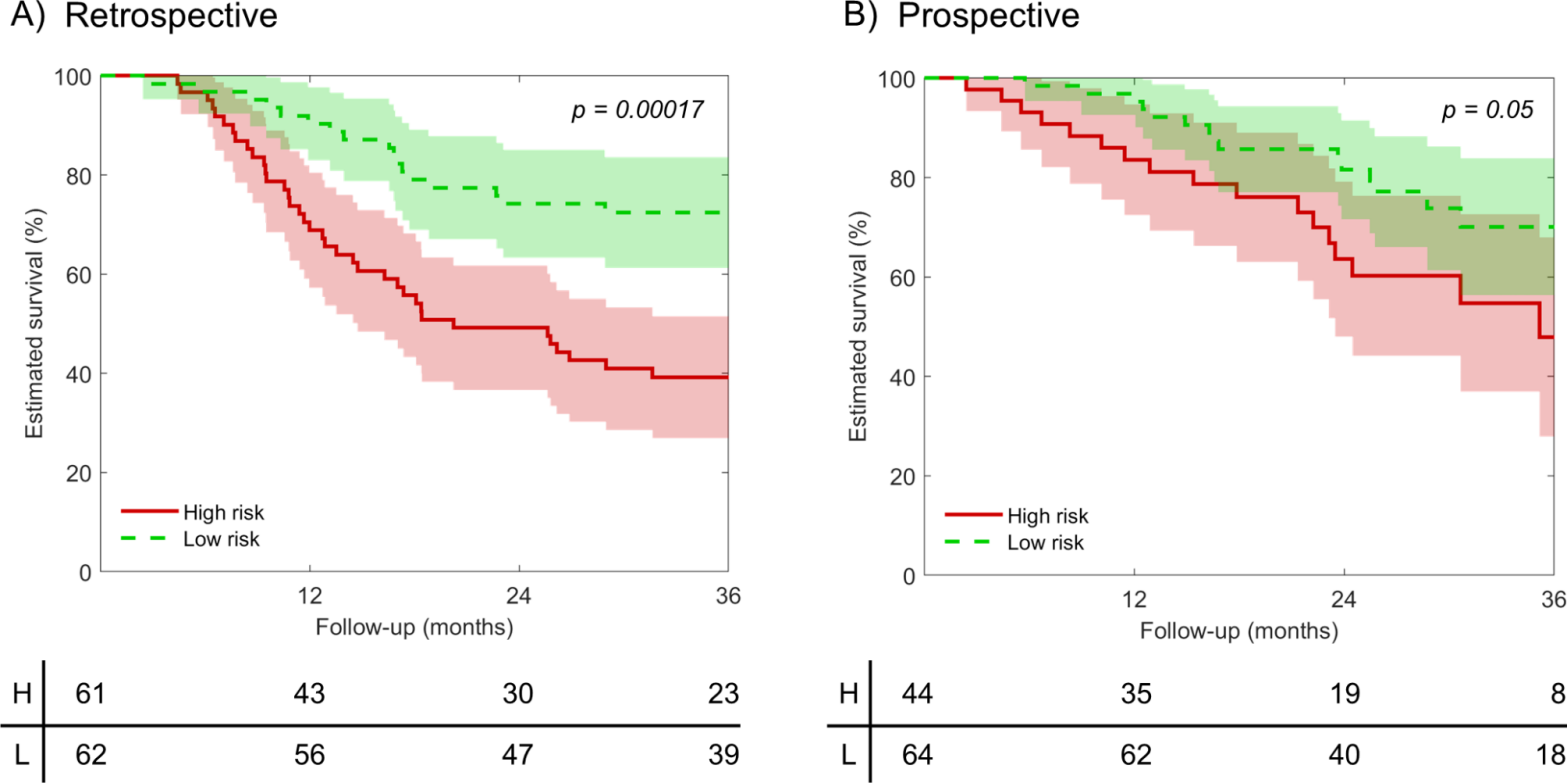
Radiomic model. (**A**) Kaplan-Meier curves for the retrospective dataset (n = 123 patients, used for model training). (**B**) Kaplan-Meier curves for the prospective dataset (n = 108 patients, used for model testing). For comparison, a follow-up time of 36 months was displayed. Shadows represent 95% confidence interval

Features selection and survival model training was performed on the retrospective dataset composed of 123 patients. From the initial 1072 radiomic features, 222 stable features (67 T1w and 155 T2w) were considered. Of these, the final 5 selected features are listed in Table 3, together with the respective means and standard deviations used for the Z-score normalization and the corresponding regression coefficients of the trained Cox model. All the 5 selected features were extracted from the waveletLLL transform of the T2w images, with 4 textural-related features and 1 first order statistics feature. The trained Cox model demonstrated a significant prognostic value of the radiomic signature for OS on the retrospective cohort: C-index 0.68 (IQR 0.66–0.70), HR 2.64 (95% CI 1.62–4.31), log-rank *p* < 0.001. The Cox model applied on the 108 prospective patients maintained for the OS a significantly prognostic value, even if less relevant, due to the shorter follow-up: C-index 0.62 (IQR 0.58–0.64), HR 2.12 (95% CI 1.04–4.47), log-rank *p* = 0.05. The Kaplan-Meier curves for the retrospective and prospective datasets (Fig. 4A and B respectively) in both cases significantly separated the high- from the low-risk groups. In both the retrospective and prospective cohorts, the radiomic signature outperformed the cTNM staging, with higher stratification capability between high- and low-risk groups and higher C-index (Supplementary figures S1 and S2).

**Table 3:** Selected features and their mean (standard deviation) and regression coefficients

Feature	Mean (std)	Coeff.
T_T2_waveletLLL_glrlm_LongRunEmphasis	1.27 (0.21)	73.07
T_T2_waveletLLL_glrlm_RunVariance	0.10 (0.08)	-59.29
T_T2_waveletLLL_glrlm_RunPercentage	0.93 (0.04)	-43.98
T_T2_waveletLLL_firstorder_Range	7.49 (3.85)	0.34
T_T2_waveletLLL_glrlm_ShortRunEmphasis	0.94 (0.03)	58.07

Mean and standard deviation of the features in the training dataset (used for the Z-score normalization) and regression coefficients of the Cox proportional hazard regression model.

### Model comparison and analysis

The radiomic prognostic model outperformed the cTNM and pTNM staging, both in terms of C-index and high/low risk patient stratification. Table 4 lists the results of the radiomic signature and the cTNM and pTNM staging in the entire prospective set of 108 patients and the prospective subset 1 (75 patients for whom the pTNM was available).

**Fig. 5:**
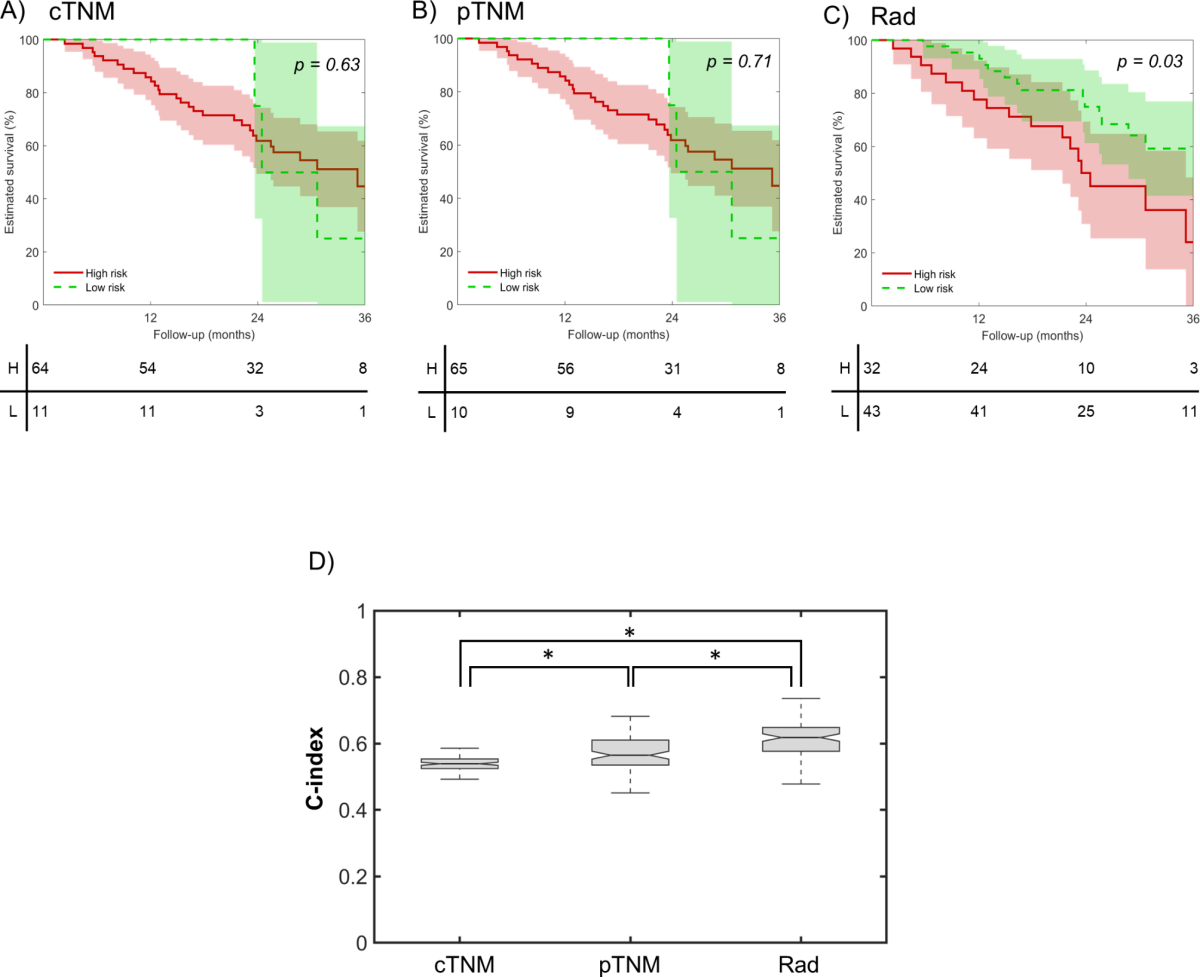
Comparison of the prognostic performance of the radiomic model with the clinical tumor-node-metastasis (cTNM) stage and pathological tumor-node-metastasis (pTNM) stage on the prospective subset 1 (n = 75 prospective patients). (**A/B**) Kaplan-Meier curves for the cTNM/pTNM, with low-risk corresponding to cTNM/pTNM stage = III and high-risk corresponding to cTNM/pTNM stage = IV. Shadows represent 95% confidence interval. (**C**) Kaplan-Meier curves for the radiomic signature. Shadows represent 95% confidence interval. (**D**) Concordance indexes (C-index) for the cTNM, pTNM and radiomic signatures. *p < 0.05 (Kruskal-Wallis with Tukey-Kramer correction)

As shown in Fig. 5, on the prospective subset 1 of 75 patients, the radiomic signature successfully separated the low/high risk group patients (log-rank *p* = 0.03), while the differences between cTNM and pTNM classes were not statistically significant (Fig. 5A and B C). Moreover, the C-index of the radiomic prognostic model was 0.62 (IQR 0.58–0.65) compared to 0.54 (IQR 0.52–0.55) of the cTNM staging and of 0.56 (IQR 0.53–0.61) of the pTNM staging (Kruskal-Wallis *p* < 0.001, with Tukey-Kramer correction) (Table 4; Fig. 5D).

**Fig. 6:**
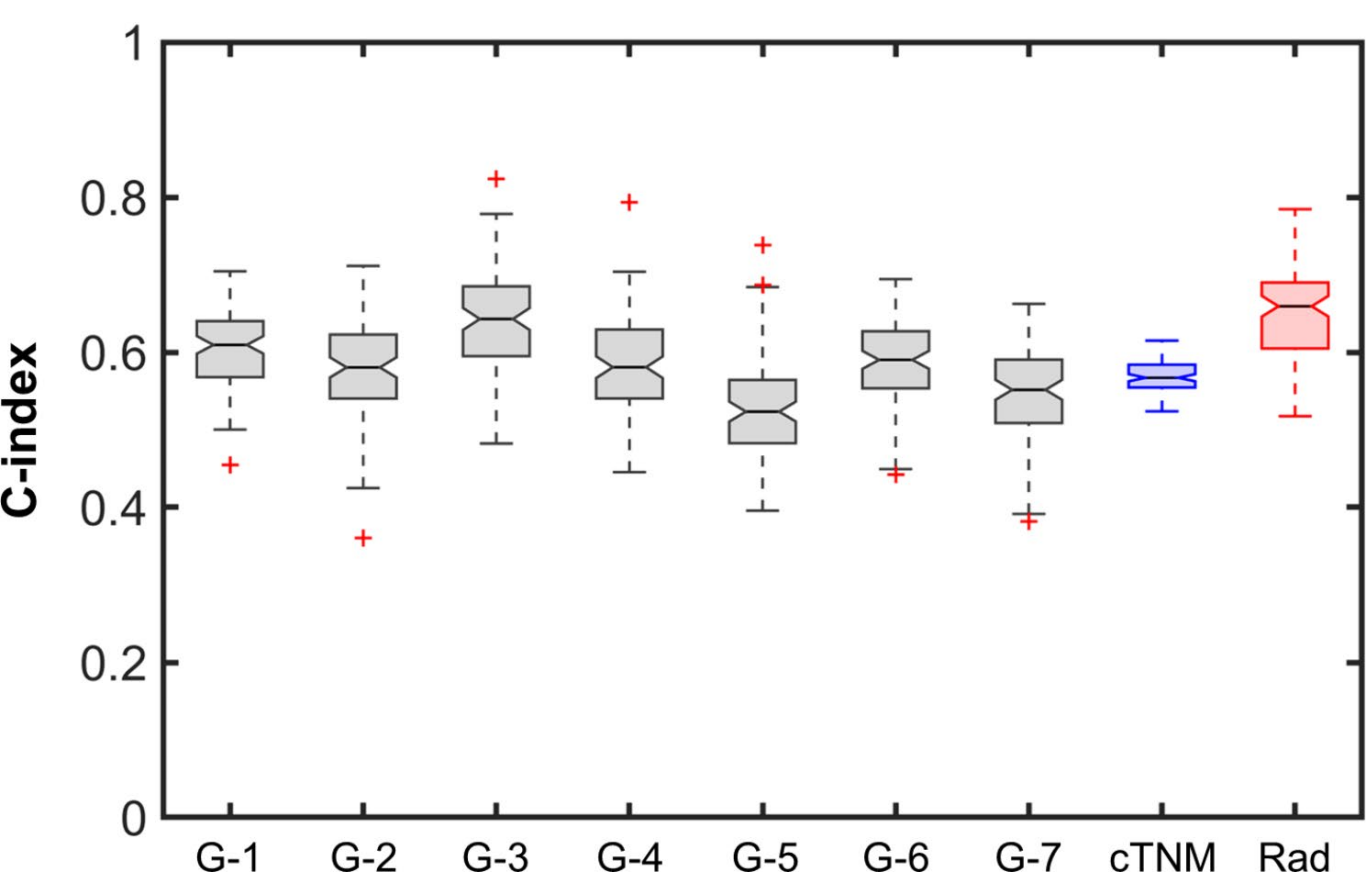
Comparison of the prognostic performance of the radiomic model with 7 prognostic genomic signatures and the clinical tumor-node-metastasis stage (cTNM) in terms of concordance index (C-index), on the prospective subset 2 (n = 90 prospective patients). Grey boxplots: C-index of the 7 genomic signatures; blue boxplot: C-index of the cTNM staging; red boxplot: C-index of the radiomic signature (Rad). The C-index of the radiomic model was significantly different (Kruskal-Wallis p < 0.001, with Tukey-Kramer correction) from that of the cTNM staging and of 6 over 7 genomic signatures (G-1, G-2, G-4, G-5, G-6 and G-7). No statistical difference was found between the C-index of the radiomic signature and G-3

From the literature survey, after filtering for the eligibility criteria, 7 gene expression prognostic signatures [48–54] (Supplementary Table 1) were identified and applied to the prospective subset 2 (n = 90 prospective patients). The prognostic performance of the radiomic model on the prospective subset 2 (patients with available gene expression) was compared with that of the 7 gene expression signatures. Figure 6 shows the C-index of the 7 genomic signatures, the cTNM staging and the radiomic signature. In particular, the radiomic model performed significantly better than 6 over 7 genomic signatures and the cTNM staging (Kruskal-Wallis *p* < 0.001, with Tukey-Kramer correction). For G-3 genomic signature the C-index (0.64, IQR 0.60–0.68) did not present statistical difference from the radiomic model (0.66, IQR 0.60–0.69).

**Fig. 7:**
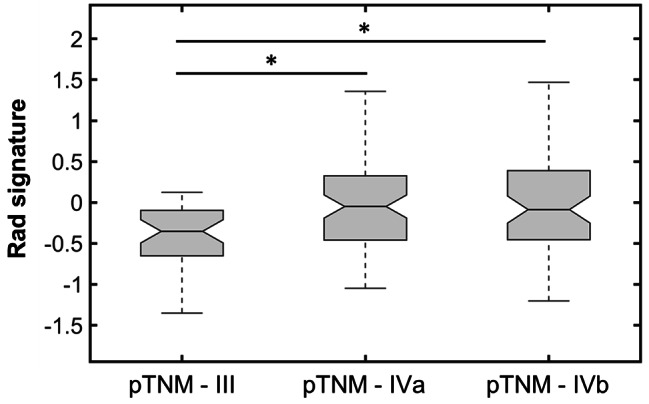
Radiomic signature distributions in patients classified according to the pathologic tumor-node-metastasis stage (pTNM = III – IVa – IVb). *p < 0.05 (Kruskal-Wallis, with Tukey-Kramer correction). n = 37 patients presented pTNM = III; n = 76 patients presented pTNM = IVa; n = 64 patients presented pTNM = IVb.

Finally, as shown in Fig. 7, the radiomic signature was significantly different between pTNM stages III and IVa/b (Kruskal-Wallis *p* < 0.001, with Tukey-Kramer correction). No significant differences in the radiomic signature distribution were observed between the pTNM stage IVa and IVb. By combining the radiomic signature and the pTNM staging in a multivariate Cox model applied to the dataset of 177 patients, we found that both are independent prognostic factors of the OS (*p* < 0.001, HR 1.85 and 1.73, respectively).

**Table 4:** Performances of radiomic, clinical and pathologic tumor-node-metastasis signatures

Dataset	Source	C-index	Log-rank HR	Log-rank p-value
**Prospective** (n = 108)	**Rad**	**0.62 (IQR 0.58–0.64)**	**2.12 (CI 1.04–4.47)**	**0.05**
	cTNM	0.55 (IQR 0.54–0.56)	1.67 (CI 0.65–4.27)	0.40
**Prospective subset 1** (n = 75)	**Rad**	**0.62 (IQR 0.58–0.65)**	**2.49 (CI 1.18–5.25)**	**0.03**
	cTNM	0.54 (IQR 0.52–0.55)	1.47 (CI 0.53–4.04)	0.63
	pTNM	0.56 (IQR 0.53–0.61)	1.40 (CI 0.49–3.91)	0.72

Rad: Radiomic signature; cTNM: clinical tumor-node-metastasis stage; pTNM: pathologic tumor-node-metastasis stage; IQR: interquartile range; CI: 95% confidence interval

## Discussion

An MRI radiomic prognostic signature, based on features extracted from T1w and T2w images of primary tumor and composed of 5 features coming from T2w images, was developed using locally advanced OCSCC from a multi-centric retrospective cohort and tested on a multi-centric prospective cohort. The radiomic signature demonstrated a significant prognostic power for OS and successfully stratified patients in low/high risk groups by Kaplan-Meier curves in both cohorts, even if the follow-up of prospective cohort was significantly shorter than that of retrospective cohort.

To date, only three studies developed MRI-radiomic prognostic signatures (based on T1w and/or T2w images) in OCSCC [32, 34, 42]. Mes et al. [32] developed a T1w-based radiomic signature on a mono-centric retrospective cohort (102 patients) and tested it on an external mono-centric retrospective cohort (76 patients). Noteworthy, in both cohorts 30–40% of stage I and II were included and the median follow-up was similar to our retrospective cohort. Despite these differences, the prognostic power and the low/high risk patient stratification performance obtained from their MRI-radiomic signature was comparable to the ones achieved herein. Wang et al. [34] developed a T2w-based radiomic signature to predict lymph node metastasis on a mono-centric retrospective cohort of 236 patients with tongue cancer (training set of 157 patients and test set of 79 patients). The combined clinical-radiomic signature was found to be an independent prognostic factor for poor OS in a multivariate Cox regression analysis (HR of 17.46). Moreover, Mossinelli et al. [42] developed several MRI-based radiomic signature for the prognosis of OS in 79 tongue cancer patients, by considering different MRI sequences. Clinical, radiomic and clinical-radiomic models were developed. When considering the radiomic models for OS, C-indexes of 0.79, 0.73, 0.72 and 0.75 were obtained with the contrast-enhanced T1, T2w, apparent diffusion coefficient map and diffusion-weighted images, respectively. From their study, the contrast-enhanced T1 sequence provided the best performance in predicting OS, while comparable results were obtained with T2w images, apparent diffusion coefficient map and diffusion-weighted images. Although the promising results, the study is limited by the low number of patients and the lack of internal and external validation. Similar to [32, 34, 42], our study demonstrated the contribution of MRI-based radiomics in OS prediction for OCSCC patients, compared to the traditionally metrics used in clinics, namely cTNM and pTNM. Besides T1w and T2w, also diffusion-weighted-based radiomics demonstrated to be useful for predicting OS, loco-regional recurrence, cause-specific mortality [42] and the histological tumor grade [70] in OCSCC patients. However, the diffusion-weighted sequence is not performed in the clinical routine, thus limiting its potential application, and was not available for the cohorts entered in the present study. Although from one side the inclusion of radiomic features from other sequences might have increased the prognostic performance of the developed radiomic model by providing complementary information, from the other side the use of standard-of-care T1w and T2w images widens its applicability, thus representing a strength of the study.

Parallel to the radiomic approach proposed herein, end-to-end radiomic-based deep learning models have been proposed (e.g., [71, 72]) and used to predict OS in HNSCC patients [73]. However, differently from the deep features, radiomic features can be linked to tissue properties, as the shape, size and texture, thus allowing for an easier interpretability of the model.

When compared to cTNM and pTNM, our radiomic model showed a higher performance in forecasting survival but the two prognostic factors (radiomics and pTNM) resulted independent predictors of outcome at multivariable analysis. Interestingly, even if evaluated in a limited number of cases (75 patients), our radiomic signature was significantly different between pathologic III and IVa/b stages suggesting that it may be useful to predict pathologic stage in patients receiving surgery. This may have an impact on treatment personalization.

The establishment of BD2Decide database provides numerous opportunities for conducting different analyses of clinical/radiomics/transcriptomics data to explore their single or combined prognostic role (see further data on [47]). As a preliminary analysis, we selected 7 gene expression prognostic signatures from the available literature and compared their performance with that of radiomic signature using the prospective dataset 2 (90 patients). Only one gene expression signature (G-3, [50]) presented, in terms of C-index, a prognostic performance comparable to that of the developed radiomic model. Also in this case, the difference in the cohorts where the signatures were developed, the anatomical subsite origin of the analyzed tumors (OCSCC versus all HNSCC) and the length of follow-up should be considered as confounding aspects. Deeper analyses and possibly a biological oriented characterization of the radiomic features will hopefully enable to better interpret these data.

The present study is not exempt from limitations. First, the small sample size and the different follow-up between the retrospective and prospective cohorts may have limited the prognostic power. Second, the prognostic performance was higher in the retrospective cohort (used for training) than in the retrospective cohort (used for validation). This can be related to the differences between the two cohorts. Cross-validation is a potential solution to address overfitting. However, model training on a retrospective cohort combined with validation on a prospective one is recommended whenever possible. Moreover, compared to cross-validation, train/validation split allows to obtained a unique prognostic model which can be applied to new external datasets. Third, only a feature selection pipeline, based on both univariate and multivariate Cox regression, was considered, based on previous studies [62]. However, as reported by Parmar et al. [31], in which 13 feature selection methods and 11 machine learning classifiers were compared in terms of prognostic performance in HNSCC patients, the feature selection method accounted for about the 14% of the total variance in the area under receiver operator characteristic curve. In future, other feature selection methods, as the least absolute shrinkage and selection operator [37, 39] or the minimum redundancy maximum relevance [31] methods will be explored as well as regularization methods for feature selection for Cox regression model [74]. Fourth, only T1w and T2w image sequences were considered because the contrast-enhanced T1 sequence was available only for a subset.

In future, the potential of radiomics in the prediction of OS in HPV+ OCSCC patients should be evaluated. Moreover, radiomic features extracted from metastatic regional lymph nodes, which were not included herein, can be considered, as done in previous works [62]. Finally, combined radiomic, clinical and genomic models can be developed to explore the additive prognostic information compared to the single models.

## Conclusion

All taken together, our results further demonstrate that MRI, which is the most used imaging modality in HNSCC patients, contains remarkable prognostic information, and can provide a non-invasive and cost-effective prognostic factor, especially given that MRI scans (and especially T1w and T2w sequences) are performed routinely in clinical practice.

Furthermore, if confirmed in other patient cohorts, the developed radiomic signature, by potentially predicting the pathologic stage in patients receiving surgery, could be applied to support the clinical decision process.

## Electronic supplementary material

Below is the link to the electronic supplementary material.

Supplementary Material 1

## Data Availability

All relevant data and materials have been included in the article and its supplementary data files. Further inquiries can be directed to the corresponding authors.

## List of Abbreviations

C-index: Concordance index
CI: Confidence interval
cTNM: Clinical tumor-node-metastasis
GLCM: Grey level co-occurrence matrix
GLRLM: Grey level run length matrix
HNSCC: Head and neck squamous cell carcinomas
HR: Hazard ratio
ICC: Intra-class correlation coefficient
IQR: Interquartile range
MRI: Magnetic resonance imaging
OCSCC: Oral cavity squamous cell carcinoma
OS: Overall survival
pTNM: Pathological tumor-node-metastasis
ROI: Region of interest
T1w: T1-weighted
T2w: T2-weighted
