# Supplementary material for: MRI-based radiomic prognostic signature for locally advanced oral cavity squamous cell carcinoma: development, testing and comparison with genomic prognostic signatures
Source: Biomark Res. 2023 Jul 16;11:69. doi: 10.1186/s40364-023-00494-5 (PMC10350277; doi:10.1186/s40364-023-00494-5)
Supplement: Supplementary file 1 — Supplementary Material 1 [file 40364_2023_494_MOESM1_ESM.docx]

**MRI-based radiomic prognostic signature for locally advanced oral cavity squamous cell carcinoma: development, testing and comparison with genomic prognostic signatures**

Anna Corti^1,*^, Loris De Cecco^2^, Stefano Cavalieri^3,4^, Deborah Lenoci^2^, Federico Pistore^3^, Giuseppina Calareso^5^, Davide Mattavelli^6^, Pim de Graaf^7,8^, C. René Leemans^8,9^, Ruud H. Brakenhoff^8,9^, Marco Ravanelli^10^, Tito Poli^11^, Lisa Licitra^3,4^, Valentina Corino^1,12^, Luca Mainardi^1^

1. Department of Electronics, Information and Bioengineering, Politecnico di Milano, Milan, Italy
2. Integrated Biology of Rare Tumors, Department of Research, Fondazione IRCCS, Istituto Nazionale dei Tumori, Milan, Italy
3. Head and Neck Medical Oncology Department, Fondazione IRCCS, Istituto Nazionale dei Tumori, Milan, Italy
4. Department of Oncology and Hemato-Oncology, Università degli studi di Milano, Milan, Italy
5. Radiology Department, Fondazione IRCCS, Istituto Nazionale dei Tumori, Milan, Italy
6. Unit of Otorhinolaryngology-Head and Neck Surgery; Department of Medical and Surgical Specialties, Radiological Sciences, and Public Health; ASST Spedali Civili of Brescia, University of Brescia, Brescia, Italy
7. Amsterdam UMC location Vrije Universiteit, Radiology and Nuclear Medicine, de Boelelaan 1117, Amsterdam, The Netherlands
8. Cancer Center Amsterdam, Imaging and Biomarkers, Amsterdam, The Netherlands
9. Amsterdam UMC location Vrije Universiteit, Otolaryngology-Head and Neck Surgery, de Boelelaan 1117, Amsterdam, The Netherlands
10. Unit of Radiology; Department of Medical and Surgical Specialties, Radiological Sciences, and Public Health; ASST Spedali Civili of Brescia, University of Brescia, Brescia, Italy
11. Maxillo-Facial Surgery Division, Head and Neck Department, University Hospital of Parma, Parma, Italy
12. Cardiotech Lab, Centro Cardiologico Monzino IRCCS, Milan, Italy

***Address for correspondence:**

Anna Corti, PhD

Department of Electronics, Information and Bioengineering, Politecnico di Milano, Milan, Italy

[anna.corti@polimi.it](mailto:anna.corti@polimi.it)

**
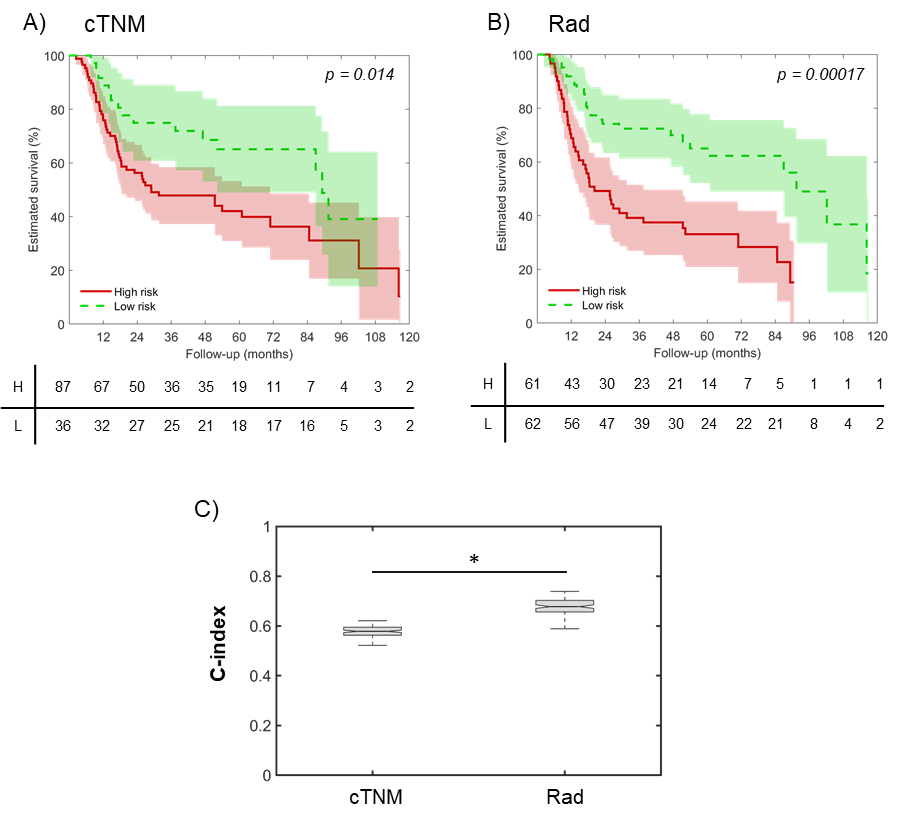
**

**Figure S1** Comparison of the prognostic performance of the radiomic model with the clinical TNM (cTNM) on the retrospective dataset (n=123 retrospective patients, used for model training). A) Kaplan-Meier curves for the cTNM. B) Kaplan-Meier curves for the radiomic signature. C) Concordance indexes for the cTNM and radiomic signatures. *p < 0.05 (Mann-Whitney).


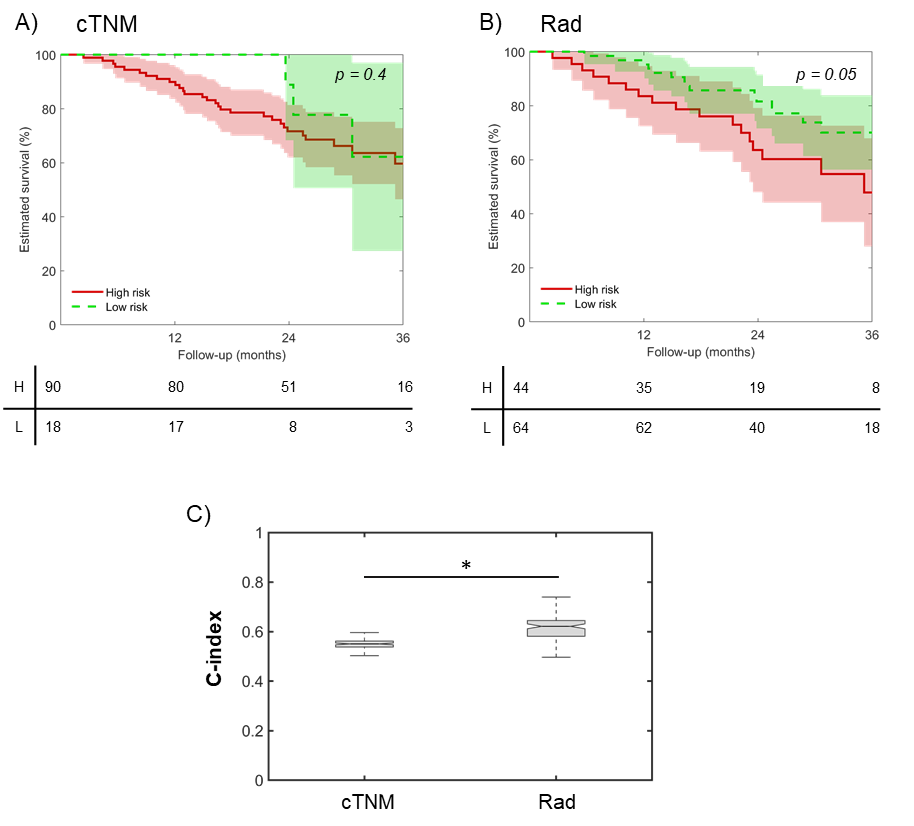


**Figure S2** Comparison of the prognostic performance of the radiomic model with the clinical TNM (cTNM) on the prospective dataset (n=108 prospective patients. A) Kaplan-Meier curves for the cTNM. B) Kaplan-Meier curves for the radiomic signature. C) Concordance indexes for the cTNM and radiomic signatures. *p < 0.05 (Mann-Whitney).

**Supplementary Table 1** Main characteristics of the prognostic selected gene expression signatures

| **ID signature** | **First author-year Reference (doi)** | **Original paper datasets** | **Number**  **Genes** |
| --- | --- | --- | --- |
| ***g1*** | Schomberg J, 2018  10.1016/j.oraloncology.2018.01.012 | 264 OSCC from TCGA | 40 |
| ***g2*** | Guo W, 2017  10.18632/oncotarget.21786 | 500 HNSCC from TCGA | 6 |
| ***g3*** | Wintergerst L, 2018  10.1002/1878-0261.12388 | 99 HNSCC from TCGA with  available information about 16q24.3  validation on 108 HNSCC LMU KKG cohort | 4 |
| ***g4*** | Lohavanichburt P, 2013  10.1158/1078-0432.CCR-12-2647 | 97 HPV negative OSCC GSE41613  Validation on 71 OSCC GSE42743 | 13 |
| ***g5*** | Liu J, 2020  10.3389/fgene.2020.00960 | 287 RT treated HNSCC from TCGA | 12 |
| ***g6*** | Wu F, 2022  10.1186/s12903-022-02193-3 | 306 OSCC from TCGA  Validation on 97 OSCC GSE41613 | 7 |
| ***g7*** | Chen Y, 2022  10.3389/fimmu.2022.922195 | 329 OSCC from TCGA  Validation on 97 OSCC GSE41613 | 5 |
